# The Use of Alcohol versus Dry Care for the Umbilical Cord in Newborns: A Systematic Review and Meta-analysis of Randomized and Non-randomized Studies

**DOI:** 10.7759/cureus.5103

**Published:** 2019-07-08

**Authors:** Hassan Al-Shehri

**Affiliations:** 1 Pediatrics, College of Medicine, Al-Imam Mohammad Ibn Saud Islamic University, Riyadh, SAU

**Keywords:** newborns, dry cord care, cord separation time, umbilical cord, neonates

## Abstract

Inadequate cord care in neonates is an important modifiable risk factor of cord stump infection, sepsis, and neonatal death, particularly in countries with limited resources. Dry cord care and alcohol 70% are commonly used in multiple developing countries. There is a need to investigate the efficacy and safety of both the cord care to achieve the best outcomes in neonates during this critical period of life. The objective of the study was to compare between dry cord care and topical application of alcohol 70% for cord care in newborn infants in terms of cord separation time (CST) as well as the incidence of omphalitis, sepsis, and neonatal mortality. The analysis was conducted up to April 2019 in MEDLINE, Cochrane Library, Embase, Scopus, and Google Scholar to include randomized clinical trials (RCTs) and quasi-experiments which investigated at least two infant groups receiving either dry cord care or alcohol 70%. Mean differences (MDs) and 95% confidence intervals (CIs) were used to analyze continuous data, while risk ratios (RRs) and 95% CIs were used to analyze dichotomous variables. A total of 13 articles were included (4967 infants, 50.35% females, six RCTs). Alcohol application was significantly associated with longer CST (MD = 1.93 days, 95% CI: 0.80, 3.06) with significant heterogeneity among studies (I^2 ^= 97%) while no significant differences were found in the risk of omphalitis. On the other hand, dry cord care was associated with the risk of foul odor at the cord/surrounding tissues (RR = 0.49, 95% CI: 0.28, 0.85) and increased risk of E-coli colonization (RR = 0.75, 95% CI: 0.57, 0.98). Dry cord care is a simple and effective way to shorten CST, particularly in countries with limited resources. However, in light of the limitations of the included studies, future RCTs with higher methodological quality are warranted. The significant heterogeneity among studies is the limitations of the included studies.

## Introduction

Newborns face one of the greatest physiological challenges during the transition from fetal to neonatal life. The newborn becomes independent once the umbilical cord is cut and the subsequent care of the cord stump represents an important stage until its detachment up to two weeks after birth [1]. This is because different types of bacteria colonize the devitalized tissues of the stump, including gram-positive bacteria, which could be followed by many enteric microorganisms [2]. More specifically, *Staphylococcus aureus* is the most predominant microorganism. Other bacteria include *S. epidermidis* and group A and B Streptococci as well as some gram-negative bacteria, such as *Escherichia coli* and* Klebsiella pneumonia* [3, 4]. Therefore, the umbilical cord remains one of the significant portals of entry of pathogenic bacteria, which might be associated with clinical signs of omphalitis [5]. The latter is defined as a bacterial infection of the umbilical stump and/or surrounding tissues occurring mostly at an average age of three days and rarely reported outside of the neonatal period.

Neonatal omphalitis is a medical emergency that requires proper management to avoid unfavorable sequelae. It may progress to systemic infection and death. Neonatal mortality attributable to omphalitis may pose a significant burden, particularly in low-resource settings. An early report indicated that the mortality rate among term and preterm neonates with omphalitis ranges between 7% and 15% [6]. More recently, it has been shown that the odds of all-cause mortality increased significantly in infants with redness of the stump that extended to the abdominal skin [7]. However, the risk of developing omphalitis has decreased remarkably with the increased rates of births in hospital, rather than home, setting. The type of employed cord care has contributed to changes in the epidemiological patterns of cord infections. Hence, the available investigations regarding incidence rates of omphalitis are scarce. Focusing on the developed countries, a recent report in the United States showed that omphalitis was diagnosed in 0.052% and 0.046% with using triple dye and dry cord care, respectively, while it was 0.1 in Canada when dry care was used [8, 9].

On the other hand, in low-income countries, cord infections occur in approximately 8% of infants born in hospitals, which increases up to 22% in infants born at home, with an estimated rate of sepsis of 2% in Pakistan [10]. Similarly, in India, omphalitis was present in 2.3% and 21.3% of hospital births and community births, respectively [11]. Indeed, the World Health Organization (WHO) Report published in 2012 revealed a stagnant state of neonatal death caused by sepsis during the period from 2000 to 2010 in India (8%) and Pakistan (9%) [12]. Such variability in the incidence of omphalitis and its consequences among different settings underscored the importance of investigating the possible modifiable risk factors.

Basically, the risk of cord stump infection increases with maternal infection, nonsterile delivery, umbilical catheterization, infants with low birth weights, prolonged labor, home delivery, and inadequate cord care [13]. The latter seems to be the most vulnerable aspect that could be optimized especially in countries with limited resources. Therefore, recommendations by the WHO for postnatal care emphasized the need for dry cord care in a hospital setting or where neonatal mortality rates are low, while chlorhexidine solution or gel should be applied to the cord for infants born in regions with high neonatal mortality rates or community births [14]. Subsequent meta-analyses based on clinical trials from India and other Asian countries revealed the relevance of chlorhexidine topical application to reduce neonatal omphalitis and mortality [15, 16]. However, its application may be associated with local dermatitis and occasionally neurotoxicity. Additionally, its efficacy may be limited against some spores and mycobacteria [17].

Therefore, there is a need to investigate the efficacy and safety of other antiseptics for cord care, considering their availability in the developing countries, to compare their use with dry care. This is to achieve the best outcomes in neonates during this critical period of life. Alcohol 70% has been used for long time in many hospital settings and it has been investigated in the literature elsewhere. In this context, this systematic review and meta-analysis assessed the difference between dry cord care and a topical application of alcohol 70% in terms of cord separation time (CST) and the resultant adverse events, including omphalitis, sepsis, and mortality.

## Materials and methods

A systematic review and meta-analysis were conducted according to the guidelines provided by the Preferred Reporting Items for Systematic reviews and Meta-analyses (PRISMA) statement [18].

Eligibility criteria

The included studies were prospective randomized clinical trials (RCTs) and non-randomized (quasi-experimental) studies published in peer-reviewed journals. Studies should allocate at least two infant groups (of any gestational age) to either dry cord care or a topical alcohol 70% regimen. The investigators should explicitly mention that the methods by which the interventions could be applied have been instructed to mothers to be performed adequately at home. In order to ensure the maximum level of internal validity of non-randomized investigations, we assured the absence of statistical clinical differences between the assigned groups at the time of allocation.

The included trials should report at least one of the primary outcomes and available in a full-text version. Studies which employed an alcohol intervention of different concentration (such as 95%) or chlorhexidine 0.5% in alcohol 70% were excluded. Additionally, retrospective studies, narrative reviews, non-English investigations were ineligible.

Types of outcome measures

The primary outcomes were CST and/or adverse events following the intervention, including omphalitis, sepsis, or mortality. If occurred, the authors should clearly present the number of infants with omphalitis rather than the mere frequency of signs of cord infection (such as redness, swelling, foul odor, etc.), because a set of signs may be reported in a single infant. The secondary outcomes included bacterial colonization, where the numbers of positive isolates of different bacterial species in the allocated infants were reported, as well as the levels of mother dissatisfaction about the provided care.

Search strategy

The following databases were searched: PubMed, Cochrane Library, Embase, Scopus, and Google Scholar. No date limit was used to optimize the search process. The search strategy was performed up to April 10, 2019 using distinct keywords and combined using the relevant Boolean operators (AND or OR). An example of the used search process is provided in Appendix 1. Besides, the bibliographies of the screened articles were searched for additional eligible trials.

Study selection and data collection

The titles and abstracts of the search results were screened by two independent authors. The records were uploaded to EndNote (version X7) and any duplicates were removed. The full-texts of eligible studies were thoroughly assessed for final inclusion. Any disagreement between authors was resolved by discussion. Microsoft Excel was used to design a specific spreadsheet for data extraction. The extracted data were as follows: 1) study data: the name of the first author, year of publication, country, study design, method of randomization for RCTs, duration of follow-up, sample size, and study groups; 2) interventions: including description of each intervention and the number of infants allocated to each group; 3) infants’ data: the number of male and female infants and mean (standard deviation [SD]) of gestational age; 4) any specific definitions of variables; 5) outcomes: mean (SD) of CST, the frequency of infants with omphalitis, sepsis, or death as well as the number of positive isolates of colonized bacteria and levels of mothers’ dissatisfaction. Countries were classified as “developed” or “developing” according to the World Economic Outlook Database of the International Monetary Fund [19].

Risk of bias

The Cochrane’s Risk of Bias Tool was used to assess the quality of included RCTs [20]. It includes the appraisal of allocation concealment, random sequence generation, blinding of the participants/personnel, blinding of outcome assessment, and other bias measurements. Data were entered and interpreted using RevMan (version 5.3, Review Manager, the Cochrane Collaboration, Oxford, United Kingdom). Two independent reviewers made their judgments and the results presented as “low risk”, “high risk” or “unclear”. Any disagreement was resolved by discussion.

Regarding non-randomized studies, we used the Newcastle-Ottawa Scale (NOS) for quality assessment [21]. This tool is used to assess the methodological quality based on three categories, including selection, comparability, and outcome and each domain is assigned a maximum score of 4, 2, and 2, respectively. Therefore, the net score for each study is 8, where the study was considered of high quality if it scored ≥ 6 and of medium quality, if it scored ≥ 4 and less than 6.

Statistical analysis

All statistical analyses were performed using the RevMan 5.3 software. Continuous variables which were reported as means (SD), such as CST, were analyzed as mean differences along with their 95% confidence intervals (CIs). On the other hand, risk ratios (RRs) and their respective 95% CIs were used for the analysis of dichotomous variables (e.g., the frequency of omphalitis, bacterial colonization, etc.). The z-statistics were used to quantify the overall effect, while the I2 test was used to investigate the degree of statistical heterogeneity among studies. A random effect model was used when the statistical heterogeneity was significant at I2 > 50%, while a fixed effect model was utilized at lower values. Subgroup analysis was performed for primary outcomes based on study design, sample size, and the economic levels of countries.

## Results

Outcomes of the search process

**Figure 1:**
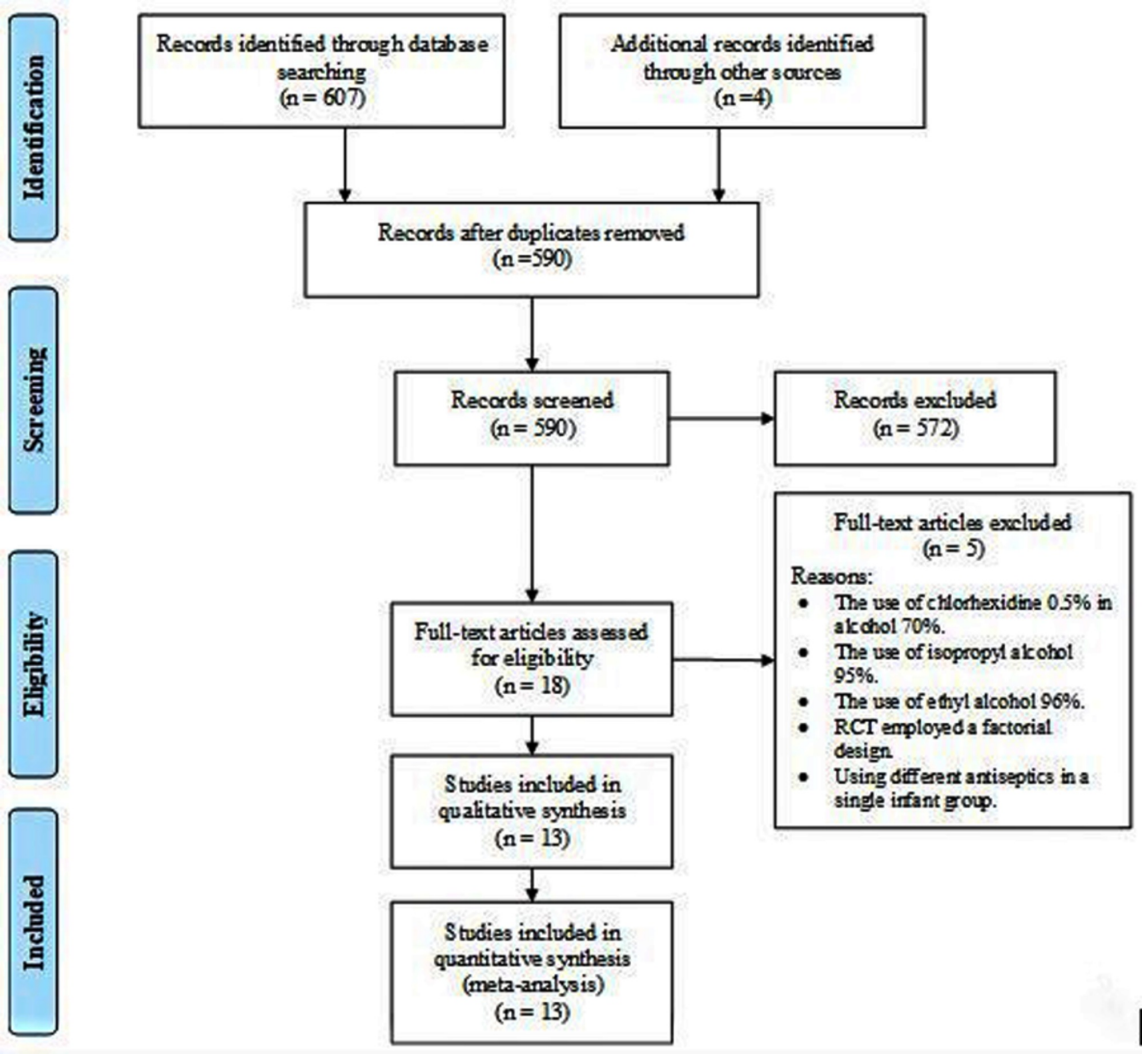
A flow diagram of the search process used in this study. RCT: Randomized clinical trial

The results of the search process are presented in Figure 1. The initial search across all databases revealed a total of 607 records, with additional four studies identified from the bibliographies of screened records. We omitted 21 duplicate records and screened the titles and abstracts of 590 search results. The full-text version of 18 articles was obtained and checked for inclusion according to the eligibility criteria. However, few articles were excluded due to the following reasons: employing different concentrations of alcohol for cord care (either 95% isopropyl alcohol or 96% ethyl alcohol), using chlorhexidine 0.5% in alcohol 70%, utilizing a factorial design of assigned interventions (which may interfere with proper interpretation), the inclusion of multiple cord regimens in a single infant group in addition to alcohol 70% [22]. Therefore, we finally included a total of 13 studies in both the qualitative and quantitative analyses.

Study characteristics

A total of 4967 infants were studied (females accounted for 50.35%). Study periods ranged between three and 20 months and sample sizes ranged between 70 and 1811 infants. The studies were conducted in five developed countries (Canada, Italy, the United States, Taiwan, and South Korea) as well as five developing countries (Thailand, Egypt, Pakistan, Iran, Turkey).

Quality assessment and risk of bias

**Figure 2:**
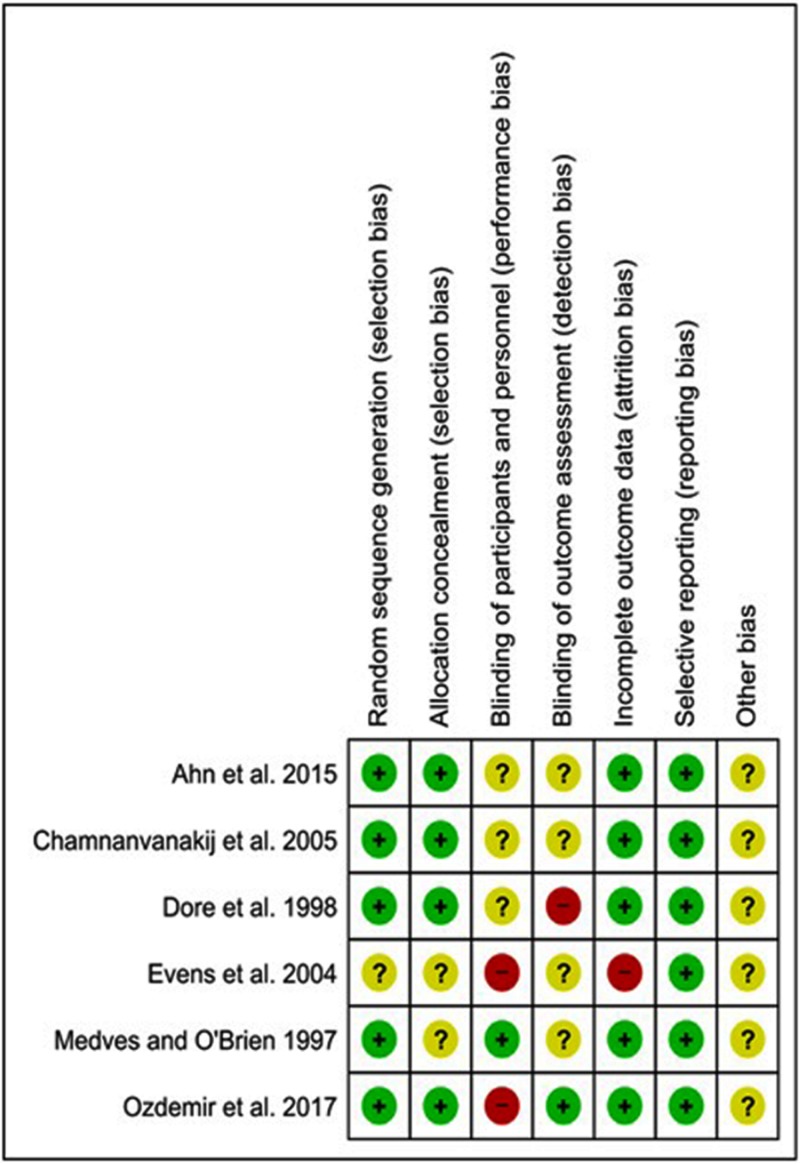
Risk of bias of the included randomized clinical trials. Red circles indicate high risk, yellow circles indicate unclear risk, and green circles indicate low risk.

The risk of bias of the included RCTs is depicted in Figure 2. Random sequence generation was performed using blocked randomization, a randomization table, consecutively numbered envelopes, and a computer software. However, Evens et al. did not clearly mention the method of infants’ randomization. Blinding of the mothers and personnel was not possible in two trials, while blinding of outcomes assessment was interrupted in a study by Dore et al., because allocation envelopes contained colored dots for the bassinette of the newborn, which ultimately indicated group allocation [23, 24].

As for quasi experiments, three studies were considered of medium quality, while the remaining studies were of high methodological quality [25-27]. The most remarkable deficient aspects related to study selection were employing a convenient sampling approach and inadequate sample selection/randomization (the first 50 infants were assigned to the dry care group and the last 50 infants to alcohol 70% group) [25, 27]. Regarding the comparability of the assigned groups, no information was available about the comparability of infants regarding gestational ages in two studies [25, 26]. In four studies, the ascertainment of infants’ interventions and the reported adverse events was not directly performed by healthcare providers, but rather only by self-reported data by the mothers as obtained one month or six weeks after birth.

Primary outcomes

**Figure 3:**
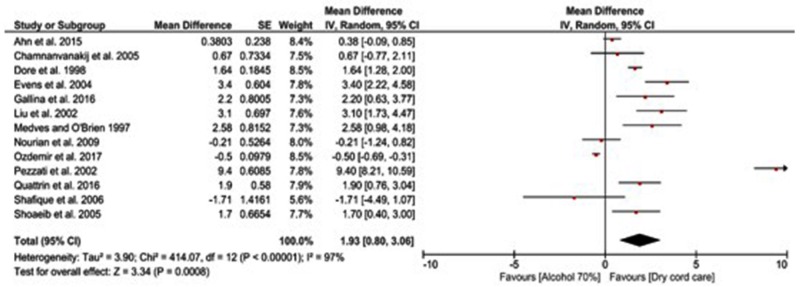
Forest plot showing the mean difference in cord separation time among infants receiving alcohol 70% or dry cord care.

The mean differences in CST are presented in Figure 3. All studies reported CST in both the dry cord care and alcohol 70% groups. Results showed that alcohol 70% regimens were significantly associated with a prolonged CST when compared to dry cord care (z = 3.34, p < 0.001) with a mean difference of 1.93 days (95% CI: 0.80, 3.06). However, there was a significant heterogeneity among all studies (I^2^ = 97%, p [heterogeneity, h] < 0.001). On subgroup analysis, the mean difference was statistically longer with alcohol 70% application among all subcategories except for developing countries and quasi experiments (Table 1).

**Table 1:** Subgroup analysis of the difference in cord separation time and the risk of omphalitis among infants under study. ^* ^Statistically significant difference at p < 0.05; ^† ^The mean difference was analyzed using the inverse variance method and a random effect model was applied; ^‡^ The risk ratio of omphalitis was analyzed using the Mantel-Haenszel method and a fixed effect model was applied. CST: Cord separation time; RCT: Randomized clinical trial.

Outcome or subgroup	CST^†^				Omphalitis^‡^			
	No. of studies	I^2 ^(%)	Effect estimate [95% CI]	P	No. of studies	I^2 ^(%)	Effect estimate [95% CI]	P
Economic status								
Developing	5	71	0.10 [-0.79, 1.00]	0.82	5	27	1.59 [0.87, 2.89]	0.13
Developed	8	97	3.05 [1.47, 4.63]	<0.001*	6	0	1.37 [0.44, 4.24]	0.59
Study design								
RCTs	6	97	1.27 [0.13, 2.41]	0.03*	4	64	0.76 [0.17, 3.35]	0.72
Quasi experiments	7	96	2.40 [-0.26, 5.07]	0.08	7	0	1.75 [0.98, 3.12]	0.06
Sample size								
Less than 150	9	84	1.58 [0.61, 2.55]	0.001*	7	0	1.89 [1.03, 3.46]	0.04*
More than 150	4	99	2.75 [0.24, 5.27]	0.03*	4	29	0.84 [0.26, 2.72]	0.77

**Figure 4:**
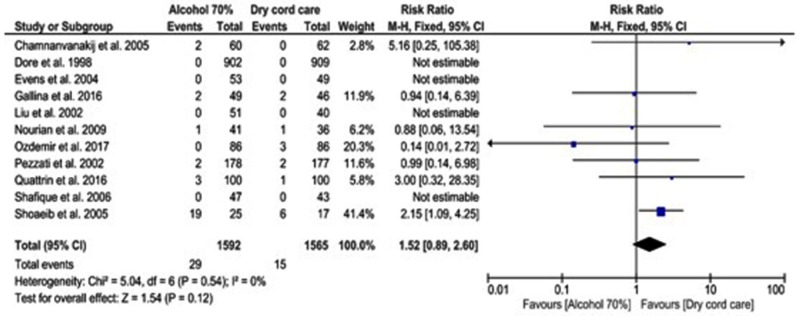
Forest plot showing the risk of omphalitis among infants receiving alcohol 70% or dry cord care.

Regarding the incidence of omphalitis, its outcomes were studied in 11 articles (3157 infants). However, RRs were not estimable in four studies since there were no reported infants with omphalitis. There was no statistical difference in the risk of omphalitis after dry cord care and the topical application of alcohol 70% (z = 1.54, p = 0.12, RR = 1.52 [0.89, 2.60]) and no significant heterogeneity was noted among the studies (I^2^ = 0%, ph = 0.54, Figure 4). The lack of significant risk of omphalitis remained apparent on subgroup analysis except for studies containing small sample sizes (less than 150 infants) where the risk of omphalitis increased with using alcohol 70% (RR = 1.89 [95% CI: 1.03, 3.46], Table 1).

**Figure 5:**
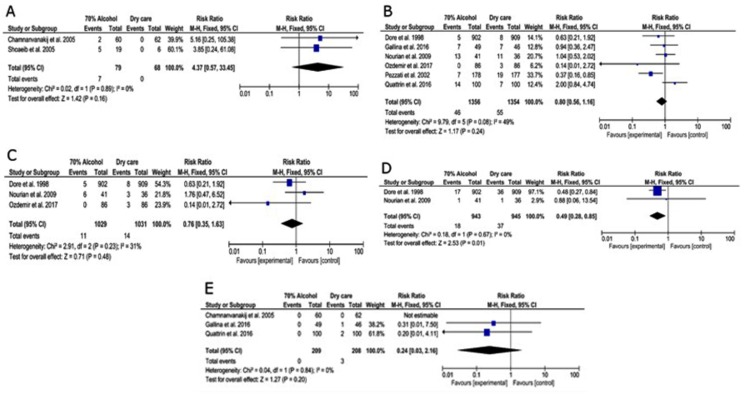
Forest plot of the difference in the risk of sepsis (A), as well as signs of cord infection, including redness (B), exudate (C), foul odor (D), and granuloma (E).

Focusing on the signs of cord infection, the presence of foul odor significantly increased with dry care when compared to alcohol 70% (RR = 0.49 [95% CI: 0.28, 0.85], p = 0.01, I^2^ = 0, Figure 5). Other signs, such as redness/bleeding, exudates, and granuloma, were similar in both types of care.

As for other adverse events, seven infants in two studies experienced neonatal sepsis (out of six studies) [27]. All infants were allocated to the topical alcohol application. However, quantitative analysis revealed insignificant differences in the incidence of sepsis between alcohol application and dry care (Figure 5). No deaths were reported among all infants in the included studies.

Secondary outcomes

Results of bacterial swabs showed that E-coli colonization was significantly higher after dry care as compared to alcohol 70% in three studies [28]. No differences were noted between both interventions in the colonization of other bacterial strains, including coagulase-negative Staphylococci, Staphylococcus aureus, and group B streptococci (Table 2). Regarding parental satisfaction about cord care, the available data were not estimable for quantitative synthesis. Instead, the qualitative evidence indicated no difference in dissatisfaction scores as consistently reported in multiple studies [23, 25, 28].

**Table 2:** The differences in bacterial colonization among infants allocated to either dry cord care or topical application of alcohol 70%. * Statistically significant difference at p < 0.05. CN: Coagulase-negative; F: Fixed effect model; R: Random effect model.

Outcome or subgroup	Number of studies	Number of infants	I^2 ^(%)	Model	Effect estimate [95% CI]	P
E-coli	3	523	0	F	0.75 [0.57, 0.98]	0.04*
CN Staph	5	554	82	R	0.82 [0.57, 1.19]	0.29
Staph. aureus	4	695	81	R	1.27 [0.45, 3.58]	0.66
B-Strept	3	523	66	R	0.67 [0.18, 2.43]	0.54

## Discussion

Cord care is an essential step in neonatal care to successfully accomplish stump separation efficiently and safely without further complications. Intra-abdominal abscesses, thrombophlebitis in the umbilical and/or portal veins, periumbilical cellulitis, peritonitis, and bowel ischemia can all occur with inadequate cord care [13]. In this study, we systematically reviewed two common methods used in areas with limited resources. Based on the findings of RCTs and quasi-experiments, our results showed that CST was significantly longer when alcohol 70% was topically applied as compared to dry cord care. Moreover, the risk of developing omphalitis and sepsis was similar with both types of care. However, according to evidence from a small number of studies, dry care was associated with higher E-coli colonization and the presence of foul odor.

In dry cord care, the stump is kept clean and dry without applying an antiseptic, a dye, or an antibiotic. In addition to its superior role over alcohol use, dry cord care was found to shorten CST when compared to chlorhexidine application (4.24 days versus 5.32 days, respectively) [5]. On the other hand, cord took off longer with alcohol use (16.9 days) when compared to other preparations, including triple dye (11.6 days) and 1% basic fuchsin (10.3 days) in an RCT conducted in a developed country [28]. Similarly, Zupan et al. found that antiseptics led to a significant prolongation of CST when compared to dry cord care in an early systematic review [29]. These findings support the effectiveness of dry care to enhance cord separation in the shortest time in healthy newborns.

In newborns, the umbilicus is the first site of bacterial colonization since it begins within the first 2-3 days of birth. Focusing on the dryness process and sloughing of the cord during its detachment, there is an increased risk of cord infection as well as the likelihood of irritation and manipulation when cord detachment is delayed [28]. Thus, it is possible that the infant may develop these signs at the cord site with an increased risk of infection as long as cord detachment is delayed with the use of alcohol, a matter which should be considered in high-risk newborns in a neonatal intensive care unit. From another perspective, delayed cord separation may pose economic and social implications. While delayed sloughing increases the frequency of domiciliary midwife visits to the home, dry cord care provides a less-expensive approach when compared to alcohol application [25]. Additionally, delayed cord separation can result in some degrees of mothers’ dissatisfaction/anxiety. In this context, it is not surprising that more rapid cord separation is preferred by families and healthcare workers.

Our results are consistent with the outcomes of a previous Cochrane systematic review, where no antiseptics (such as alcohol) were advantageous in reducing the risk of omphalitis compared with dry care [16]. In addition, the authors concluded that alcohol use was associated with a significant reduction of *Enterococcus coli *colonization based on pooling the results of two studies (RR = 0.73, 95% CI: 0.58 to 0.92). Likewise, Zupan et al. emphasized the trend of reduced bacterial colonization with using topical antibiotics and antiseptics as compared to dry care [29]. However, interestingly, there was no difference in the colonization of Staphylococcus aureus, which is the main cause of neonatal omphalitis. Actually, the relationship between basic bacterial colonization and the confirmed incidence of omphalitis remains unclear [9]. This is supported by the high frequency of positive bacterial isolates in many studies with no or few reported cases of clinical cord infection [24, 26, 28].

The inclusion of low-quality trials, particularly those from developing countries, may lead to misleading outcomes although their reported results were similar to those studies conducted in developed countries (Table 2). Continuing with limitations in study designs, blinding of mothers to the allocated treatment was not possible as they were involved in the care process and were instructed to report any warning signs of cord infection. Further, we tried to minimize the risk of bias that may emerge from the intentional allocation of infants in non-randomized trials by ensuring the lack of difference between study groups at the time of allocation.

Other limitations could be apparent in the current study. We included studies recruiting healthy infants and thus the outcomes could not be estimable to high-risk infants. In some instances, healthcare workers were not able to directly observe the warning signs of infection in neonates but rather the mothers were instructed to do so at home [25]. This might affect the real incidence rates of adverse events. Additionally, we have included English language publications and thus some non-English trials might have escaped inclusion. Finally, the use of potentially harmful substances according to some cultural beliefs in the dry cord group may have confounded the results.

In conclusion, there is significant evidence that supports implementing dry cord care for effective and rapid separation of the cord in newborn infants as compared to the use of alcohol 70%. However, both kinds of care have no unfavorable effects on the risk of omphalitis, sepsis and mortality. Given that these findings were based on randomized and non-randomized studies of low to medium quality evidence, future trials are warranted, considering well-designed methodological approaches and including a large number of infants. Additionally, specific emphasis should be placed on the association between mere cord colonization and the confirmed incidence of omphalitis. Finally, other interventions, especially chlorhexidine, should be extensively investigated in comparison to dry care in a systematic manner.

## Conclusions

There is significant evidence that supports implementing dry cord care for effective and rapid separation of the cord in newborn infants as compared to the use of alcohol 70%. However, both kinds of care have no unfavorable effects on the risk of omphalitis, sepsis, and mortality. Given that these findings were based on randomized and non-randomized studies of low to medium quality evidence, future trials are warranted, considering well-designed methodological approaches and including a large number of infants. Additionally, specific emphasis should be placed on the association between mere cord colonization and the confirmed incidence of omphalitis. Finally, other interventions, especially chlorhexidine, should be extensively investigated in comparison to dry care in a systematic manner.

## Appendices

**Appendix 1: **The used search strategy used in PubMed

#1 “umbilical cord” OR “umbilicus”

#2 “newborn” OR “infant”

#3 #1 AND #2

#4 “alcohol 70%” OR “alcohol”

#5 “dry” OR “dry cord care”

#6 #4 AND #5

#7 #3 AND #6

#8 randomized controlled trial [Publication Type] OR controlled clinical trial [Publication Type]

#9 randomized [Title/Abstract]

#10 randomly [Title/Abstract]

#11 trial [Title/Abstract]

#12 groups [Title/Abstract]

#13 quasi [Title/Abstract]

#14 #9 OR #10 OR #11 OR #12 OR #13
